# Department of Practice of Medicine

**Published:** 1893-09

**Authors:** 

**Affiliations:** Medical Department University of Texas, Galveston


### Current Medical Literature.

### DEPARTMENT OF PRACTICE OF MEDICINE.

Conducted by Professor Allen J. Smith, A. M., M. D., Medical Department University of Texas, Galveston.

THE TREATMENT OF CHOLERA BY HYPODERMOCLYSIS AND ENTEROCLYSIS (*American Journal of Medical Sciences*, July, 1893).—Dr. Judson Doland discussing this subject, speaks of the treatment in the three stages, (1) premonitory stage, (2) evacuant or stage of collapse, and (3) the stage of reaction. In the first of these the writer counsels rest in bed, protection from cold, the simplest character of food, such as boiled milk, well-boiled rice and milk toast. The administration of an acid, hydrochloric acid (30 or 40 drops to a tumbler of water for the adult) or sulphuric acid (20 drops in four ounces of water) as recommended by Dr. Roland G. Curtin, is advised because of the inability of the cholera spirilla to grow in acid media. In the stage of collapse on account of the enormous loss of fluid, and the serious consequence of such loss to the blood and muscles, the subcutaneous injection of a solution of 0.6 per cent. (or two small teaspoonfuls) of sodium chloride in a quart of hot water (distilled and sterilized), with the addition of several ounces of brandy perhaps, supplies most thoroughly and simply the indications.

For the employment of hypodermoclysis an ordinary fountain syringe, with a small sized aspirating needle and canula is best suited. The operation should be performed in either flank between the ribs and the crest of the ilium, or on the inner aspect of the thighs. The entire apparatus should be sterilized; the skin about to be punctured should be first well washed with soap and water, then with alcohol, next with ether and afterward saturated with a 1:500 solution of mercury bichloride. The first injection to an adult should be one or two quarts; for an adolescent one or two pints, and for an infant, one half pint. The solution should have a temperature of 110° F., in the reservoir, which should lose five degrees or thereabout in traversing the tube to the needle. The liquid should be introduced slowly, the hydrostatic pressure being easily regulated by raising or lowering the reservoir of the syringe. Twenty or thirty minutes should be consumed in introducing one quart of the liquid, dispersion of the fluid under the skin being aided by massage only in cases where rapid absorption is demanded. In favorable cases unaided, absorption takes places in from twenty to forty-five minutes, but in some cases it requires several hours; and the rapidity of absorption is held as of some prognostic value. Inorder to counteract the processes set up by the cholera micro-organisms in the intestine, where the stomach is retentive, full doses of such medicaments as salol or salicylate of sodium should be admidistered by the mouth. Where this is impossible, because of the irritability of the gastric mucous membrane, or even aside from such circumstances, enteroclysis, the introduction of fluid into the intestinal canal by the rectum, is a valuable method of medicinal administration. Dr. Doland has proved to his entire satisfaction that liquids may be passed with greater or less ease through the ileo-cæcal valve from the lower into the upper intestine; and details a number of experiments performed upon the living as well as the dead subject to verify his assertion. In enteroclysis the author recommends the use of a two per cent. solution of tannic acid in water (about three teaspoonfuls to the pint), at a temperature of 110° F. Of this, two quarts for an adult, one for an adolescent, and a pint for a child should constitute the dosage. The solution should be introduced quite slowly, the author suggesting the employment of a lavage tube of soft rubber, with rounded end, a diameter of a fourth of an inch, with an outlet about half an inch from the extremity, and a second one on the opposite side of the tube several inches further back. The tube should be well oiled, warmed and slowly inserted into the rectum by a rotary and forward movement for a distance of ten inches. At least ten minutes should be consumed introducing a quart of liquid, and its retention should be sought for. In severe cases enteroclysis may be repeated as often as every four hours. If retained the fluid inhibits the growth of the cholera spirilla, supplies heat, and replaces a ceramount of fluid lost through the processes of the disease. If it be not retained, it at least serves to wash out from the intestine much material, which if absorbed, would be noxious. In order to supply the necessary heat in this algid stage, Dr. Doland recommends the above measures, the hot plunge-bath repeated as often as desired, hot air introduced beneath the bedclothes, hot water-bags, hot bricks, or even the use of a water-bed filled with hot water as suggested by Dr. F. X. Dercum. As stimulants the author recommends hypodermic doses of brandy repeated as often as every hour. He recommends that the choleraic matters in the stomach be washed out by lavage with the hot tannic acid solution used in enteroclysis. The only nourishment permitted should be peptonized or sterilized milk, about two ounces every two hours. Koumyss or carbonated milk may be substituted if this is not well borne. Iced champagne may be given in small quantities. Carbonated distilled water, or the table-waters, such as Apollinaris, with hydrochloric acid as suggested, may be permitted. In the stage of reaction the quantity of liquid food and liquids may be increased. The acid should be continued. If suppression of urine continues into this stage, it would be an indication for continuance of hypodermoclysis at interval of eight hours. Pepsin may be now administered in order to encourage perfect digestion, along with the hydrochloric acid. The food in uncomplicated cases should gradually pass from the liquid form to more solid substances as soft-boiled eggs, milk-soaked bread, well-boiled rice, junket, etc. Where the typhoid stage remains, the hypodermoclysis should be repeated once or twice daily; and strychnine (gr. $\frac{1}{40}$.) and quinine (gr. iii) may be administered thrice daily along with the pepsin and hydrochloric acid.

---

TREATMENT OF TYPHOID FEVER BY THYMIC ACID.—Dr. E. E. Wible (*Pittsburg Medical Review*, March, 1893) reports that during the autumn and winter of 1892 he treated forty-eight consecutive cases of typhoid fever by the administration of five grains of thymol (thymic acid) every three hours until convalescence was established or the patient's temperature approached the normal. The drug may be administered internally either in emulsion or dissolved in alcohol, but is best given in pill form, using soap or glucose as an excipient. Of these forty-eight cases there were but three deaths, one moribund on admission, dying within twenty-four hours after admission; a second admitted during the fourth week of illness in a typhoid state and having had no medical attention during this time. Excluding the one admitted to hospital in a dying condition, the rate of mortality is but a small fraction over four per cent. The length of the course of the disease was also favorably influenced by the thymol treatment, the average number of days the patients remained in hospital being less than thirty. The average of the highest temperature in each case was $103\frac{4}{5}°$ F. The highest temperature of any case was $105\frac{3}{5}°$ F. in the axilla, and this elevation remained for five days before it gradually lowered. The cold wet pack was employed to lower the temperature. The patients were all males between the ages of 19 and 55 years; their average was 26 years. Three cases were complicated by intestinal hemorrhage, but these all recovered. Other complications that occurred among this series were: two cases of parotiditis; three cases of suppurative otitis; one of phlebitis, and one of periostitis. One of the cases of parotiditis occurred in one of the fatal cases.

Dr. Wible appends to his paper the following rules for the guidance of nurses in the treatment of typhoid fever patients:

1. Give each patient five grains of thymol every three hours.

2. Give ten grains of salicylate of bismuth every three hours, when there are more than three to four stools in twenty-four hours.

3. Give to each patient two quarts of skimmed milk every twenty-four hours.

4. Give cracked ice and ice-water freely, allowing from three to eight quarts in twenty-four hours.

5. If the diarrhœa is not marked, beef-tea or chicken broth may be given once or twice a day.

6. If the diarrhœa is excessive, give nothing but milk and water.

7. When the patient's temperature rises to or above 103° F., give sponge baths every half hour or apply the cold wet pack until the temperature is reduced below 103° F.

8. For abdominal pain or tympanites apply turpentine stupes.

9. Cleanse and disinfect the mouth with a solution of boric acid.

10. Disinfect the stools and urine with a thick solution of the chloride of lime or a five per cent. solution of carbolic acid.

11. Give no solid food until the evening temperature has been normal ten days.

12. Patients may be allowed to sit up for a short time about the end of the first week of convalescence.

---

A NEW TREATMENT FOR TUBERCULOSIS.—Samuel G. Dixon (*Times and Register*, April 29, 1893) reports excellent results in the treatment of a case of skin tuberculosis by the endemic administration of tannin and urea. The use of these substances as well as creatin, uric acid and other members of the amido group was suggested to the writer by the rarity of coincidence of tuberculosis and the gouty state, which latter is sought to be approximated by the introduction of these substances.

---

ICE IN THE TREATMENT OF ACUTE PNEUMONIA.—Thomas J. Mays, of Philadelphia, publishes in the *Medical News*, June 24, 1893, the reports of fifty cases of acute pneumonitis treated by application of ice to the chest over the affected areas, with a view of overcoming the inflammatory process. These cases occurred in his own practice, or were collected by the writer from literature, or from personal reports of the attending physicians. Two of the number, or four per cent. died; but it must be added that one of these fatal cases was a very unpromising one, a sufferer from chonic lead poisoning and intemperate, while the other one probably died from an acute exacerbation of an old attack. The ages of the patients ranged from six and one-half months to seventy-four years. In some instances the ice was applied over a period of several weeks without any untoward results becoming manifest. The author states his belief in the resolving power of the cold upon the inflammation, acting mainly by causing capillary contraction in the affected tissues. The application of the ice-bag acts beneficially upon the symptomatology of the affection, remarkably relieving the pain, dyspnœa, cough and expectoration, as well as very decidedly influencing the temperature. Firandt (*London Lancet*, Aug. 10, 1892) has recently reported

106 cases of pneumonia treated with ice application, with but three deaths (2.82 per cent). These, with the above, made a series of 156 cases with five deaths, (or a death rate of 3.2 per cent. Ordinarily it is not uncommon to meet with an epidemic of pneumonia whose death ratè is at least ten times greater than this; and the rate of most hospitals for a long series of years is over 20 per cent., the old method of treatment being pursued.

---

SHAKING PALSY.—Charles L. Dana, of New York, (*N. Y. Medical Journal*, June 10, 1893,) thus summarizes a study of this affection, based upon the observation and post mortem examination of several patients: Paralysis agitans is characterized by a central vascularzation of the spinal cord, a diffuse interstitial sclerosis starting from the blood vessels and pia. This affects in particular the central and anterior portions of the gray matter and lateral columns, leading in later stages to cell degeneration, leptomeningitis and some peripheral sclerosis; there is sometimes degenerative neuritis of the peripheral nerves, and chronic myositis; the cerebral cortex and the basal ganglia and the cerebellum, and, in fact, the brain as a whole, is but slightly and only secondarily involved; this chronic irritative process is due to a toxine which circulates in the blood, and may be of endogenous, and perhaps glandular origin; the disease process first affects the end brushes surrounding the anterior horns and causes their degeneration; and it finally impairs the anatomical structure of the motor and vaso-motor secretory cells, causing degeneration and atrophy of them to some extent.

In the matter of treatment the author regards opium as of most service, after rest. Salicylate of sodium and salol often secure excellent results, and the writer believes the proper remedy must be sought for in the shape of some antitoxine which will counteract the poison circulating in the nervous centers. The toxine supposed to underly this disease process is, it is true, but a hypothetical one as yet; yet the analogies to such conditions as gout and rheumatism, the known value of opium and the action of opium upon the glandular functions, as well as the nature of changes in the nervous system render it probable that there does exist such a poison, which may moreover be a product of pathological glandular activity.

---

YELLOW FEVER—Dr. Joseph Jones, (*St. Louis Med. and Sur. Journal, May, 1893,*) continuing his remarks upon yellow fever, an abstract of which appeared in this journal for the month of ———— of the present year, states that while many of the most striking phenomena of the disease, as chills and fever and collapse, must be attributed to disordered vascular inervation, the true point of commencement of the malady is in the alteration of the blood. Nevertheless the nervous system, both the cerebro-spinal and sympathetic, from its distribution and widely reaching functions, also becomes in its disarrangement one of the prominent factors in the affection. During the active stages of yellow fever serious changes take place in the organs and tissues, especially in the kidneys, heart and liver, degenerative in nature, oil and albuminiod granular matter appearing within the cells and in the tissue spores. The blood shows a marked diminution of the fibrinous elements, an increase of fatty matter and an accumulation of biliary and urinary constituents. Upon the hæmic alteration and upon the direct irritation and structural alteration of the gastric mucous membrane by the yellow fever poison, and the biliary and excrementitious substance in the blood is due to the symptomatic "black vomit." The chief causes of death in yellow fever appear to be the direct action of the febrile poison on the blood and nervous system, depressing and disarranging the functions of the latter, and rendering the former unfit for the proper nutrition of the tissues; the suppression or alteration of certain functions, as those of the kidneys and liver, with the retention in the blood of the substances normally eliminated by them; structural alterations and consequent loss of power of the heart, and profuse hemorrhages from the stomach and bowels.

Yellow fever differs essentially from malaria in that in the latter the red blood cells, in the former the albumen of the blood suffers to the greatest extent, in that in the former the temperature changes follow a definite course and are never repeated in an uncomplicated case, while in the latter they at regular intervals and may be indefinitely reproduced; in that yellow fever as a rule attacks but once, whereas malarial fever does not exempt from further attack, but seems to create a predisposition; in that convalescence in yellow fever is generally brief, the changes of blood and organs, as spleen and liver, in malaria being often profound and long continued; in that in yellow fever the liver is yellow and contains numerous oil globules; in malaria it is dark and loaded with pigment particles; the spleen is comparatively unaffected in yellow fever, but enlarged and altered in malaria; the heart and kidneys are softened and full of oil droplets in yellow fever, but comparatively unchanged in malaria; the urine in yellow fever is almost always albuminous and full of bile and casts in yellow fever, but rarely much affected in case of malaria.

Yellow fever is self limited and usualy occurs but once in the life time of an individual. The urine in yellow fever is persistently acid, its specific gravity does not vary much from the normal. At first it is normal in color, clearness and quantity, but soon it becomes turbid and yellow from the presence of bile, epithelium, casts and granular matter, and contains albumen in considerable quantity.

---

SALYICLATES IN THE TREATMENT OF PLEURISY WITH EFFUSION—George Dock, of Ann Arbor, Mich. (*Therapeutic Gazette, Feb. 15, 1893*), concludes an article upon the treatment of pleurisy with effusion as follows:

1. Salicylic acid and its salts are among the most effectual agents is the treatment of pleurisy with effusion.

2. In effective doses the remedy is harmless, and with proper selection of the preparation and care in administration causes little or no discomfort to the patient.

3. Salicylates act most promptly in pleurisies with recent or long standing effusion, but are effective in simple dry pleurisy, and often act favorably in secondary pieurisy.

4. There is no evidence that they are useful in suppurative cases.

5. The drug acts as a diuretic, but may have an effect on the pathological process, or on the cause of the disease.

6. Salicylates have a more marked action in pleurisy than have the diuretics commonly so called.

7. "The duration of the treatment with salicylic preparations is less than with diuretics, common salt or roborant medication." (Engster,)

8. The remedy can be used at the earliest period and favorably affects all symptoms.

9. The drug may be given in the form of the acid, or any of its salts, in doses of a drachm of the former, or one to two drachms of a salt daily. In ordinary cases it is not necessary to give the large doses, and sixty to ninety grains of sodium salicylate or salol daily may be considered full beginning doses, to be diminished one-third or one-half after the effect is manifest.

10. The ordinary precautions must be observed in giving the drugs, and during their administration the total amount of urine should be measured daily.

---

THE TREATMENT OF HÆMOPTYSIS.—Dr. Fr. Eklund, of Stockholm (*Therapeutic Gazette*, Feb., 1893), severely condemns the common practice of giving cold drinks and small bits of ice to the patient with hæmoptysis to swallow. He believes that the irritation of the ends of the pneumogastric nerve in the mucous membrane by the cold must result in paroxysms of coughing, as is the experience with many phthisical patients, and that the cold tends to contract the vessels of the stomach and vicinity, leading to higher pressure in the pulmonary area. Instead, he advises lukewarm, mucilaginous drinks. He also advises against the popular practice of giving large amounts of salt dissolved in water, since the fragility of the vessels is increased upon absorption of the sodium chloride and fluid. He suggests that a small ice bag be placed over the bleeding spot, and that quinine be administered as follows:

℞ Sulphate of quinine..................ʒj

Extract of ergot................gr. xxx

M. Make into forty pills, and take one or two pills twice or three times a day.

Or the following may be given:

℞ Fluid extract of hamamelis ..........ʒij

Fluid extract of cinchona ............ʒij

Extract of liquorice................ʒijss

Distilled water.......................Oj

Shake thoroughly, and take a dessertspoonful to a tablespoonful every two or three hours.

He advises that in young persons lead acetate be withheld, for fear of colic; in older persons, this remedy may be of service, but should be given in combination with morphine.

